# Computational geometric tools for quantitative comparison of locomotory behavior

**DOI:** 10.1038/s41598-019-52300-8

**Published:** 2019-11-12

**Authors:** Matthew T. Stamps, Soo Go, Ajay S. Mathuru

**Affiliations:** 1 Yale-NUS College, Singapore, Singapore; 2 Institute of Molecular and Cell Biology (IMCB), Singapore, Singapore; 3 Department of Physiology, Yong Loo Lin School of Medicine (YLL), National University of Singapore, Singapore, Singapore

**Keywords:** Neuroscience, Applied mathematics

## Abstract

A fundamental challenge for behavioral neuroscientists is to accurately quantify (dis)similarities in animal behavior without excluding inherent variability present between individuals. We explored two new applications of curve and shape alignment techniques to address this issue. As a proof-of-concept we applied these methods to compare normal or alarmed behavior in pairs of medaka (*Oryzias latipes*). The curve alignment method we call Behavioral Distortion Distance (BDD) revealed that alarmed fish display less predictable swimming over time, even if individuals incorporate the same action patterns like immobility, sudden changes in swimming trajectory, or changing their position in the water column. The Conformal Spatiotemporal Distance (CSD) technique on the other hand revealed that, in spite of the unpredictability, alarmed individuals exhibit lower variability in overall swim patterns, possibly accounting for the widely held notion of “stereotypy” in alarm responses. More generally, we propose that these new applications of established computational geometric techniques are useful in combination to represent, compare, and quantify complex behaviors consisting of common action patterns that differ in duration, sequence, or frequency.

## Introduction

Quantitative comparison of the behavior of animals is a key element of neuroscience. As important as this is, even the most comprehensive ethograms fail to capture the inherent variability in the behaviors of individuals, whether they consist of simple or complex sets of actions^1–4^. This is further compounded by the error-prone nature of subjective assessment during human observations. A pertinent issue for behavioral studies, therefore, is the development of appropriate methods for the quantitative comparison of behaviors between two animals or a single individual under two or more conditions.

Researchers typically quantify parameters (such as the average speed or average height climbed) based on expert knowledge of the ethology of that species or the kinematics of a behavior. These measures are useful, yet they can be inadequate as descriptors of behavior for a variety of reasons. One notable reason is that they are gross approximations of the behavioral action being measured. Averaging of observational data may not be representative of the kinematics and can be confounded by what has been described elsewhere as “the failure of averaging^2^”. Furthermore, there can be considerable variability in the execution of even those behaviors that are considered “stereotypical” or instinctive. As elaborated at great length by Schleidt in 1974, individual variability can exist in the form of “incompleteness of the elements” that make up an innate behavior or in the form of changes in the characteristics of those “elements” such as their duration, sequencing, or frequency within a given episode of executing the behavior^5^. Trait variability between individuals such as locomotor handedness is also influenced by alellic differences^6^. Consequently, the above mentioned gross approximations are at best imperfect representations of the behavior that neither capture nor do justice to the intra and inter subject variability^7^. Another reason measures fail to be adequate descriptors of behavior is that even if specific parameters are defined as relevant in a behavioral readout, they may not be available when examining a new species or when studying previously uncategorized behaviors. Quantification of infrequent events (such as episodes of immobility or jumps) is useful, but also somewhat unsatisfactory as a measure since it omits and obscures a large portion of the behavioral data. In an ideal scenario, an entire behavioral episode is represented in as complete a form as possible during quantification.

Studies over the past few years have addressed these (or related) issues by applying tools from computational geometry, computer vision, and machine learning to describe behaviors^8^. These methods have proved effective in describing animal behavior more comprehensively. For example, in examining the entire behavioral repertoire of the hydra^9^, for automated annotation of behavior in fruit flies^10^, for discovering generative rules governing *Drosophila* locomotion^11^, for identifying the temporal features that explain spontaneous swimming behavior in worms^12^, for identifying the dynamics of shoaling in fish^13^, and for detecting sub-second modules in mice behavior^14^. Such studies are providing new insights like the continuity between behavioral states^15^, between the generative rules of locomotion of vertebrates and invertebrates^11^, and objective means for comparison of new data-sets to perform spatiotemporal mapping of posture of non-stereotyped actions^7^. Here, we report on two new applications of computational geometric methods that can be applied broadly for the representation and quantitative analysis of dissimilarity in the locomotory behavior of pairs of animals moving freely in a two-dimensional space. We applied these methods to compare swimming behavior of pairs of fish.

The first among these methods applies a technique for automated curve alignment developed by Sebastian *et al*.^16^ that can be efficiently calculated with a method called *dynamic time warping*^17^. Dynamic time warping has been applied widely to time series analysis^18,19^ as well as the study of biological phenomena, such as speech patterns^20^ and cardiac filling phases^21^. In recent years, others have employed techniques based on dynamic time warping to examine and compare temporal patterns and acoustic features in bottle-nosed dolphins^22^, whales^23^, and songbirds^24^. The same technique has also been adapted to compare locomotion and infer postures in humans^25,26^. Motivated by a classic result in differential geometry called the Frenet-Serret Formula^27^, we introduce the notion of a *behavior curve* associated to an animal’s trajectory and derive a measure that we call the Behavioral Distortion Distance (BDD). This can be efficiently calculated via dynamic time warping to provide meaningful quantitative comparisons for entire episodes of locomotion.

The second application we considered examines the spatiotemporal kinetics encoded in heat maps. When comparing heat maps between animals that represent the time spent at each location in an arena, it is not useful to look at a straightforward average since hot spots from different animals will average out and disappear. Instead, one must first align the heat maps so that corresponding hot spots coincide in order to reveal if there are common patterns and similarities. We implemented a well-established algorithm^28–32^ used previously in cortical cartography^33,34^, for finding conformal (angle preserving) alignments between surfaces – a heat map can be interpreted as a surface in 3-dimensional space where different intensities correspond to different heights in the *z*-direction – that uses discrete geometric objects called *circle packings*. We call the resulting measure between heat maps the Conformal Spatiotemporal Distance (CSD).

As a proof-of-concept, we asked if these methods can quantitatively distinguish normal swimming behavior from a behavior described as “alarmed” in fish^35^. We compared medaka fish from a preexisting data-set that had been exposed either to an alarm substance or to a control^36^. Though the species where such a phenomenon has been described have expanded considerably^37–40^ since its discovery in minnows approximately 80 years ago^41^, alarm behavior to conspecific injury has been mainly studied in the Ostariophysi species of fish^42,43^. Even among Ostariophysi, species differ in the expression of alarmed behavior. Many different parameters, in captivity and in the wild, have been described for different fish including a change in inter-individual distance in shoals, freezing, darting, change in the vertical position in a water body, etc.^44–48^. As such, it is difficult to predict what an alarmed behavior in response to the chemosensory alarm substance will look like in a species where this has not been described before, or when they have multiple defensive behaviors^49^. We therefore chose as our test case a single published study (at the time we started this project) from one of the authors describing an alarm response in medaka^36^. We asked if the individuals in the two conditions (alarmed and normal) were distinguishable without us defining what action sequence or locomotory pattern constitutes an alarmed state.

We found that BDD and CSD can differentiate between alarmed and normal states. The BDD between most pairs of alarmed individuals were also high, which suggests that swimming trajectories can become so unpredictable when medaka are alarmed that they may not match a second individual responding to the same stimulus. On the other hand, the CSD between most pairs of alarmed individuals were low, highlighting the existence of a discernible spatiotemporal pattern in spite of low predictability in their swimming trajectory. Overall, our results suggest that BDD and CSD together are useful aides to represent long episodes of swimming behavior. Our results also indicate that these computational geometric methods can be applied more universally for quantitative comparison of locomotion of any kind, in any animal.

## Results

The preexisting data-set we used for this report recorded the swimming motion of 36 medaka over a 10-minute interval^36^. The observational tanks were featureless small rectangular tanks with dimensions 20 cm × 12 cm × 5 cm (*L* × *H* × *W*). While the tanks restricted movement compared to the animals’ natural habitat, they allowed multiple degrees of freedom in swimming motion and can be accepted as reasonably “free” spaces for motion. In particular, the fish could swim in any manner across the tank approximately six to seven body lengths long, four body lengths deep, and one and a half body lengths wide. Half of the fish were exposed to an alarm substance (*Schreckstoff* or SS) at the same time point (minute 2 in a 10 minute observation), while the other half were exposed to a control substance (tank water or NSS). Mathuru^36^ discovered a statistically significant difference in the average speed of the experimental subjects (SS) compared to the control subjects (NSS) during the post-stimulus (2–10 minute) time interval (Mann-Whitney U = 49.0, p = 0.0003) and a statistically significant difference in intervals of immobility (defined as displacement less than 3 mm in 1 second by the experimenter; Mann-Whitney U = 13.0, p = 0.000002).

Here, we explored the use of two additional measures that rely on notions of “distance” between the swimming patterns of pairs of subjects using the tracked data generated from the automated tracker in the previous study^36^. The BDD or the Behavioral Distortion Distance, defined in the methods section, measures the differences in local locomotory behavior (independent of location and relative time) whereas the CSD or the Conformal Spatiotemporal Distance, also defined in the methods section, measures the spatiotemporal location pattern of a subject over a specified duration. For all of the results listed in this article, BDD refers to the behavioral distortion distance indexed by speed and curvature, or BDD_Θ_ for Θ = {*s*, *κ*} in the notation of the Methods section.

### Analysis of pairwise differences in behavior

**Figure 1:**
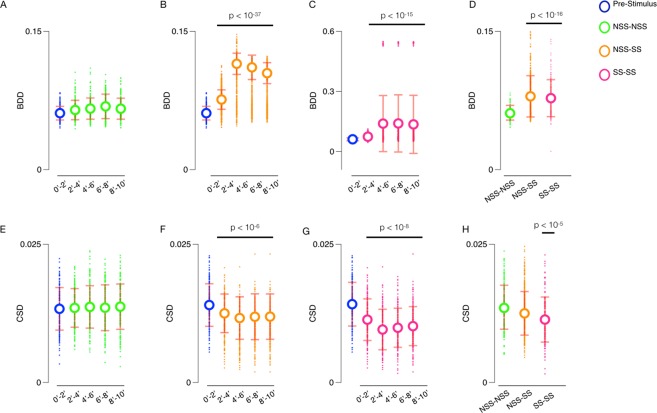
Panels (A–C) show the distributions, means, and standard deviations of BDD for pairs of fish over five 2 minute time intervals broken down into three categories: pairs of control fish (NSS-NSS; Green), mixed pairs with one control and one alarmed fish (NSS-SS; Orange), and pairs of alarmed fish (SS-SS; Purple). Panel (D) shows the distribution, mean, and standard deviation of BDD for pairs in each category over the entire post-stimulus time interval. Panels (E–G) show the distributions, means, and standard deviations of CSD for all pairs of fish over five 2-minute time intervals broken down, again, into three categories: pairs of control fish (NSS-NSS; Green), mixed pairs with one control and one alarmed fish (NSS-SS; Orange), and pairs of alarmed fish (SS-SS; Purple). Panel (H) shows the distribution, mean, and standard deviation of CSD for pairs in each category over the entire post-stimulus time interval. The distributions over the pre-stimulus interval (minutes 0 to 2) are all plotted in blue.

**Figure 2:**
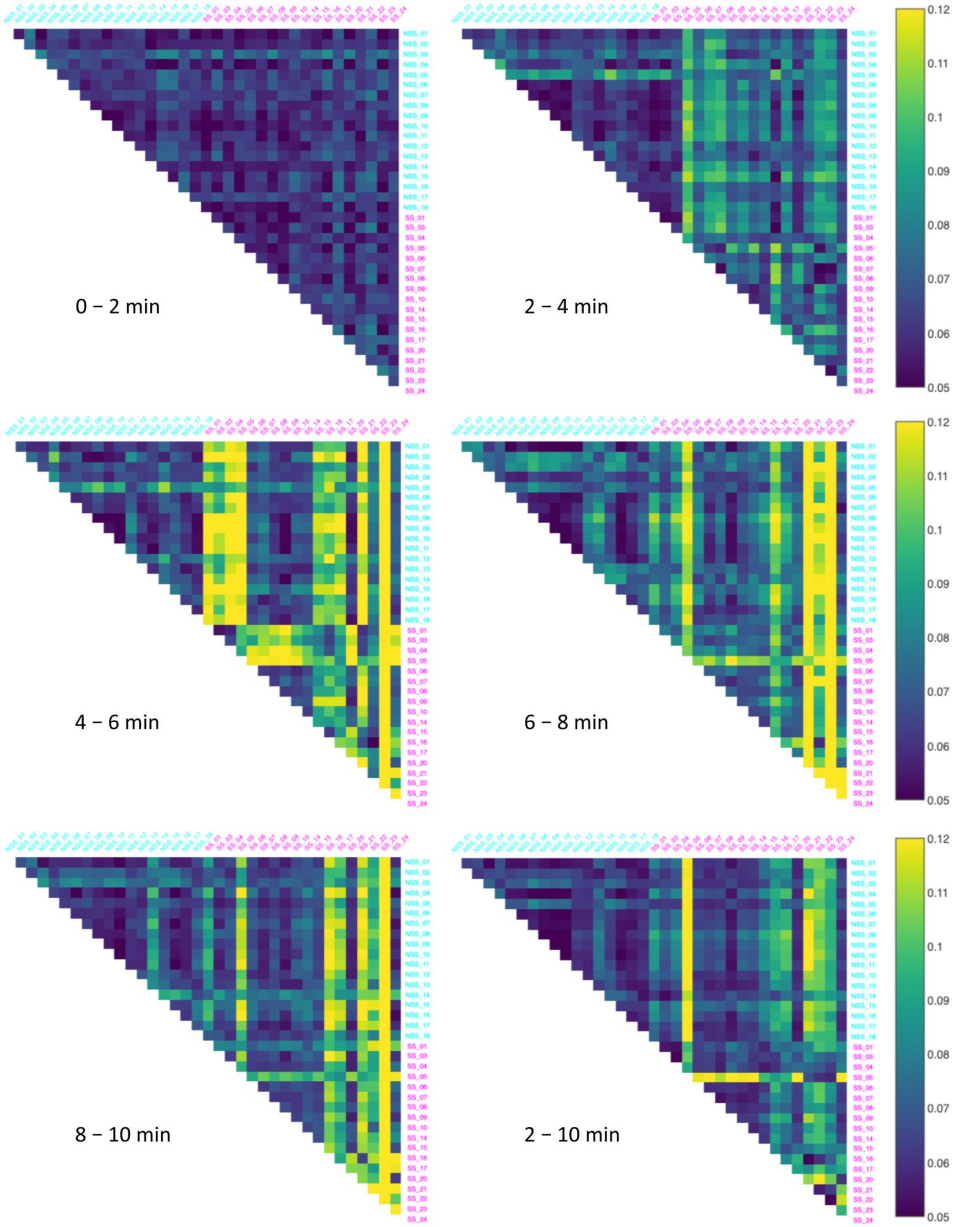
The Behavioral Distortion Distances (BDD) between all pairs of the thirty-six fish in the dataset over five 2-minute time intervals and the entire post-stimulus time interval. The alarmed (SS) individuals are labeled magenta and the control (NSS) individuals are labeled cyan.

**Figure 3:**
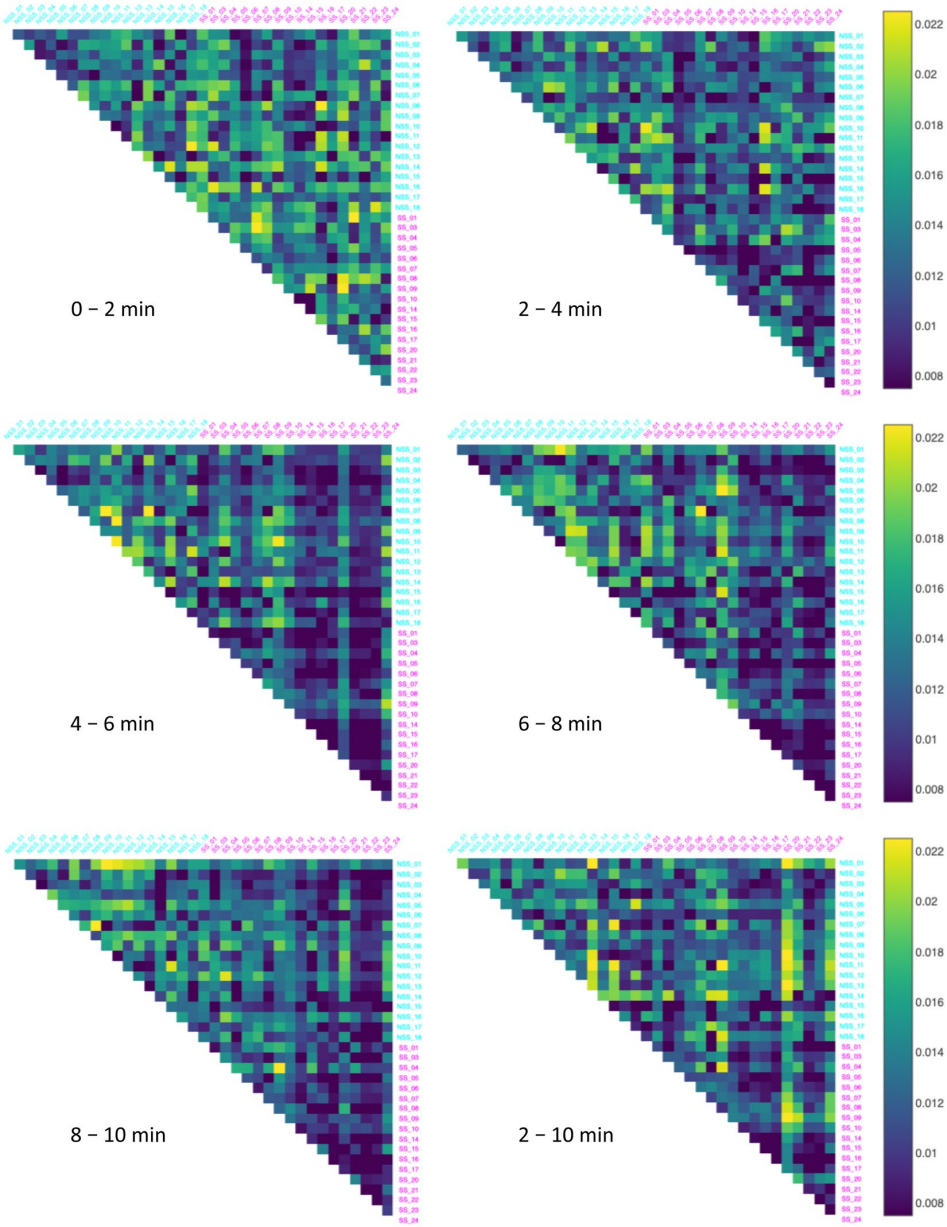
The Conformal Spatiotemporal Distances (CSD) between all pairs of the thirty-six fish in the dataset over five 2-minute time intervals and the entire post-stimulus time interval. The alarmed (SS) individuals are labeled magenta and the control (NSS) individuals are labeled cyan.

The BDD and CSD between all pairs of the 36 test subjects over five 2-minute time intervals and the entire post-stimulus (minutes 2 to 10) time interval are illustrated in Fig. 1. The pairs of subjects are split into three categories: pairs of control subjects (NSS-NSS), mixed pairs with one control and one experimental subject (NSS-SS), and pairs of experimental subjects (SS-SS). The BDD and CSD for each pair of subjects and specified duration are illustrated in Figs 2 and 3, respectively, and the summary statistics are given in Tables 1 and 2.

**Table 1:** The means and standard deviations for the BDD between pairs of subjects over five 2-minute time intervals and the post-stimulus time interval, broken down into three categories: pairs of control subjects (NSS-NSS), mixed pairs with one control and one experimental subject (NSS-SS), and pairs of experimental subjects (SS-SS).

Time Interval (minutes)	Mean				Standard Deviation			
	All	NSS-NSS	NSS-SS	SS-SS	All	NSS-NSS	NSS-SS	SS-SS
0–2	0.061	0.062	0.061	0.061	0.0076	0.0074	0.0076	0.0076
2–4	0.073	0.065	0.076	0.074	0.0145	0.0105	0.0146	0.0145
4–6	0.109	0.066	0.115	0.140	0.1057	0.0115	0.1055	0.1394
6–8	0.108	0.069	0.111	0.140	0.1071	0.0132	0.1068	0.1422
8–10	0.103	0.066	0.105	0.136	0.1080	0.0114	0.1077	0.1445
2–10	0.075	0.061	0.080	0.078	0.0207	0.0085	0.0223	0.0200

**Table 2:** The means and standard deviations for the CSD between pairs of subjects over five 2-minute time intervals and the post-stimulus time interval, broken down into three categories: pairs of control subjects (NSS-NSS), mixed pairs with one control and one experimental subject (NSS-SS), and pairs of experimental subjects (SS-SS).

Time Interval (minutes)	Mean				Standard Deviation			
	All	NSS-NSS	NSS-SS	SS-SS	All	NSS-NSS	NSS-SS	SS-SS
0–2	0.0139	0.0133	0.0140	0.0142	0.00388	0.00383	0.00394	0.00375
2–4	0.0125	0.0135	0.0125	0.0114	0.00373	0.00348	0.00371	0.00374
4–6	0.0116	0.0137	0.0117	0.00957	0.00394	0.00382	0.00361	0.00365
6–8	0.0118	0.0135	0.0119	0.0099	0.00410	0.00413	0.00396	0.00352
8–10	0.0119	0.0137	0.0119	0.0102	0.00392	0.00409	0.00364	0.00352
2–10	0.0127	0.0135	0.0129	0.0114	0.00408	0.00409	0.00393	0.00406

Figure 1 reveals that while the pre-stimulus interval BDD are similar between the three categories, the mean BDD between NSS-SS pairs and between SS-SS pairs are both larger than the mean BDD in the pre-stimulus period, as well as the mean BDD between NSS-NSS pairs for each of the post-stimulus time intervals. In a comparable manner, the mean CSD between NSS-SS pairs and between SS-SS pairs are both smaller than the mean CSD in each of the post-stimulus time intervals, while it remains the same between NSS-NSS pairs. Figure 2 provides a colored visual aide to compare BDD between all pairs, while Table 1 summarizes this information. It shows that the mean BDD ranged between 0.061 and 0.069 for NSS-NSS pairs for both the pre- and post-stimulus intervals. However, the ranges increased in the post-stimulus period to between 0.074 and 0.141 for NSS-SS pairs and SS-SS pairs. In a similar manner, Fig. 3 provides a colored visual aide to compare CSD between all pairs and Table 2 and shows that the mean CSD between NSS-NSS pairs over the pre- and post-stimulus time intervals ranged between 0.0133 and 0.0137, while, for NSS-SS pairs and SS-SS pairs, the range decreased from approximately 0.014 to between 0.0095 and 0.0125. Kruskal-Wallis H statistical tests confirmed that the differences over the five 2-minute time intervals for NSS-NSS pairs for both the BDD (Fig. 1A, H-statistic = 6.759, *p* = 0.149) and the CSD (Fig. 1E, H-statistic = 0.897, *p* = 0.925) were statistically insignificant. However, they did reveal statistically significant differences for the BDD between NSS-SS pairs (Fig. 1B, H-statistic = 387.579, *p* = 1.34 × 10^−82^), between SS-SS pairs (Fig. 1C, H-statistic = 259.470, *p* = 5.92 × 10^−55^); and the CSD between NSS-SS pairs (Fig. 1F, H-statistic = 76.781, *p* = 8.37 × 10^−16^), between SS-SS pairs (Fig. 1G, H-statistic = 124.940, *p* = 4.70 × 10^−26^). This suggests that the BDD and CSD over each of the specified 2-minute time intervals throughout the duration of the assay (10-minute) is unchanging when control substance or tank water is introduced, but statistically significant differences emerge when one or both members of the pair are exposed to *Schreckstoff*.

To evaluate if the exposure to *Shcreckstoff* is the reason for the differences in the BDD and CSD measures above, we performed pairwise comparisons between the pre-stimulus time interval (0–2 minutes) and each of the four 2-minute post-stimulus intervals via Wilcoxon signed-rank T tests. This revealed statistically significant increases in the BDD between NSS-SS pairs (Fig. 1B, all T-statistics <4600, all *p*-values < 10^−37^) and the BDD between SS-SS pairs (Fig. 1C, all T-statistics <1400, all *p*-values < 10^−15^). Statistically significant decreases in the CSD between NSS-SS pairs (Fig. 1F, all T-statistics <18000, all *p*-values < 10^−6^) and between SS-SS pairs (Fig. 1G, all T-statistics <2600, all *p*-values < 10^−8^) were also detected. Therefore, the BDD increases while the CSD decreases between pairs of medaka after stimulus delivery when at least one individual is exposed to *Schreckstoff*. Next, we also conducted pairwise comparisons of the distributions of BDD and CSD between the three categories of pairs over the entire post-stimulus time interval. In Fig. 1D, the mean BDD between NSS-SS pairs and SS-SS pairs are both larger than the mean BDD between NSS-NSS pairs. To verify that these differences are statistically significant, we conducted Mann-Whitney U tests between the distributions of BDD for NSS-NSS pairs and NSS-SS pairs (Fig. 1D, U-statistic = 10730.0, *p* = 1.49 × 10^−23^) and NSS-NSS pairs and SS-SS pairs (Fig. 1D, U-statistic = 5282.0, *p* = 1.05 × 10^−16^). In Fig. 1H, the mean CSD between SS-SS pairs is less than that between NSS-SS pairs, which is less than that between NSS-NSS pairs. Again, to verify that these differences are statistically significant, we conducted Mann-Whitney U tests between the distributions of CSD for NSS-NSS pairs and SS-SS pairs (Fig. 1H, U-statistic = 15126.0, *p* = 9.84 × 10^−6^) and for NSS-SS pairs and SS-SS pairs (Fig. 1H, U-statistic = 30470.0, *p* = 5.24 × 10^−5^). The Mann-Whitney U tests did not reveal statistically significant differences between the distributions of BDD for NSS-SS pairs and SS-SS pairs (Fig. 1D, U-statistic = 25437.0, *p* = 0.64), nor did they reveal statistically significant differences between the distributions of CSD for NSS-NSS pairs and NSS-SS pairs (Fig. 1H, U-statistic = 26729.5, *p* = 0.17). Therefore, this suggests that the BDD detected different levels of variation in behavior between pairs of control subjects and pairs with at least one experimental subject. On the other hand, CSD detected different levels of variation in behavior between pairs of experimental subjects and pairs with at least one control subject over the post-stimulus time interval. Moreover, the variations in behavior measured by BDD between pairs of experimental subjects were as large as those between mixed pairs with one control and one experimental subject.

We also compared the distributions of BDD and CSD between the three categories of pairs and the set of all pairs (again over the entire post-stimulus time interval). Mann-Whitney U tests revealed that the mean BDD between NSS-NSS pairs is significantly lower than that of all pairs (U-statistic = 68674.5, *p* = 3.33 × 10^−16^) and the mean BDD between NSS-SS pairs is significantly higher than that of all pairs (U-statistic = 87351.5, *p* = 2.63 × 10^−4^). Additionally, Mann-Whitney U tests revealed the mean CSD between SS-SS pairs is significantly lower than that of all pairs (U-statistic = 57300.5, *p* = 2.85 × 10^−4^). The Mann-Whitney U tests did not reveal statistically significant differences between the distributions of BDD for SS-SS pairs and all pairs, nor did they reveal significant differences between the distributions of CSD for NSS-NSS pairs and all pairs, nor NSS-SS pairs and all pairs. Therefore, the variations in behavior detected by BDD between NSS-NSS pairs and NSS-SS pairs are significant compared to the set of all pairs, not just to SS-SS pairs and each other. Similarly, the variations in behavior detected by CSD between SS-SS pairs are significant compared to the set of all pairs, not just to the other two categories of pairs (NSS-NSS and NSS-SS).

Finally, to further illustrate that the differences reported in the previous paragraphs are unlikely to be the results of random fluctuations, we conducted an additional analysis based on randomized (also known as permutation) tests^50^. For this, we compared the mean BDD between NSS-SS pairs to a distribution of mean BDD between randomly relabeled pairs. Specifically, we uniformly sampled 100000 of the 9075135300 ways to divide the 36 test subjects into two 18-element subsets and calculated the mean BDD between the subsets in each of those partitions. Then, we compared the mean BDD between the NSS-SS partition to the distribution of mean BDD for randomized partitions. The corresponding distribution statistics, standard scores, and *p*-values are listed in Table 3.

**Table 3:** The mean BDD between NSS-SS pairs compared to the distribution of mean BDD between pairs from randomly generated partitions of the 36 assay subjects into two 18-element subsets, with corresponding standard scores and *p*-values, over each of the specified time intervals.

Time Interval (minutes)	Randomized Partitions		NSS-SS Partition		
	Distribution Mean	Distribution Std. Dev.	Mean BDD	Z-score	P-value
0–2	0.061	0.00023	0.061	−0.48	0.628
2–4	0.073	0.00046	0.076	6.93	4.21 × 10^−12^
4–6	0.109	0.00069	0.115	8.42	3.80 × 10^−17^
6–8	0.108	0.00047	0.111	6.67	2.64 × 10^−11^
8–10	0.103	0.00042	0.105	4.28	0.000019
2–10	0.075	0.00054	0.079	9.24	2.36 × 10^−20^

Together, these results and statistical analyses suggest that the variability in dynamics and trajectory increase, while the variability in spatiotemporal preference decreases, when the pair of medaka being compared contains at least one alarmed individual.

### Individual variability and classification

Following our analyses of pairwise comparisons, we asked if BDD and CSD could yield meaningful information about individual behavioral episodes, without direct comparison to other episodes in a larger data-set. One approach for this is to measure the distance between a subject and itself over different (non-overlapping) time intervals. This is not practical for CSD, since that method only captures meaningful information over extended periods of time. We calculated the mean BDD from each subject to itself over 1000 non-overlapping sub-intervals of the post-stimulus time interval, sampled uniformly at random. The resulting values, which we call the Intra-Individual BDD (IIBDD) for each subject, are listed in Supplementary Table 1. A Mann-Whitney U test showed that the mean IIBDD of experimental subjects is significantly higher than that of control subjects (U-statistic = 23.5, *p*-value = 1.25 × 10^−5^). Therefore, the variation in behavior measured by BDD from an experimental subject to itself is, on average, significantly larger than that from a control subject to itself.

This led us to ask if IIBDD could be used to classify normal and alarmed behaviour by itself. We conducted three logistic regressions on the thirty-six test subjects using average speed, and immobility, the parameters used in the previous report, and IIBDD. The logistic regression for average speed had an intercept of 4.449 and a coefficient of −0.168 (*χ*^2^ = 15.562, *p*-value = 7.98 × 10^−5^); the logistic regression for immobility had an intercept of −3.005 and a coefficient of 0.302 (*χ*^2^ = 30.659, *p*-value = 3.08 × 10^−8^); and the logistic regression for IIBDD had an intercept of −20.680 and a coefficient of 244.250 (*χ*^2^ = 25.494, *p*-value = 4.44 × 10^−7^). The data used for these regressions are in Supplementary Table 1 and the corresponding classifications are given in Table 4. The logistic regressions show that all three parameters can be used to classify normal and alarmed behavior, with duration of immobility and IIBDD exhibiting better goodness of fit than average speed. Therefore, IIBDD allows for classification of a new animal’s behavior as alarmed or normal if the new experiment replicates the conditions of the original experiment.

**Table 4:** Logistic regression results for classifying normal (NSS) versus alarmed (SS) behavior given the average speed, duration of immobility, and Intra-Individual BDD (IIBDD), respectively. Regression outputs less than 0.5 are classified as NSS and those greater than 0.5 are classified as SS. Incorrect classifications are indicated with bold font.

Subject	Speed Classification	Immobility Classification	IIBDD Classification
NSS 01	NSS (0.189)	NSS (0.069)	NSS (0.065)
NSS 02	NSS (0.222)	NSS (0.074)	**SS** (**0**.**754**)
NSS 03	**SS** (**0**.**764**)	**SS** (**0**.**658**)	NSS (0.437)
NSS 04	NSS (0.218)	NSS (0.137)	NSS (0.152)
NSS 05	**SS** (**0**.**760**)	**SS** (**0**.**804**)	NSS (0.411)
NSS 06	NSS (0.209)	NSS (0.045)	NSS (0.063)
NSS 07	NSS (0.309)	NSS (0.080)	NSS (0.038)
NSS 08	NSS (0.029)	NSS (0.056)	NSS (0.009)
NSS 09	NSS (0.141)	NSS (0.052)	NSS (0.032)
NSS 10	NSS (0.251)	NSS (0.074)	NSS (0.009)
NSS 11	NSS (0.147)	NSS (0.045)	NSS (0.006)
NSS 12	**SS** (**0**.**593**)	NSS (0.120)	NSS (0.447)
NSS 13	**SS** (**0**.**591**)	NSS (0.347)	NSS (0.257)
NSS 14	**SS** (**0**.**523**)	NSS (0.267)	**SS** (**0**.**806**)
NSS 15	NSS (0.119)	NSS (0.049)	NSS (0.031)
NSS 16	NSS (0.108)	NSS (0.074)	NSS (0.193)
NSS 17	NSS (0.332)	NSS (0.056)	NSS (0.085)
NSS 18	NSS (0.118)	NSS (0.056)	NSS (0.297)
SS 01	SS (0.507)	SS (0.998)	SS (0.988)
SS 03	SS (0.866)	SS (1.000)	SS (0.889)
SS 04	**NSS** (**0**.**380**)	SS (0.791)	SS (0.966)
SS 05	SS (0.864)	SS (0.998)	SS (1.000)
SS 06	SS (0.960)	SS (1.000)	SS (0.613)
SS 07	SS (0.636)	SS (0.874)	SS (0.847)
SS 08	**NSS** (**0**.**405**)	SS (0.952)	**NSS** (**0**.**323**)
SS 09	SS (0.816)	SS (0.927)	SS (0.518)
SS 10	**NSS** (**0**.**140**)	**NSS** (**0**.**188**)	SS (0.534)
SS 14	SS (0.757)	**NSS** (**0**.**493**)	SS (0.635)
SS 15	SS (0.782)	SS (0.998)	SS (0.993)
SS 16	SS (0.872)	SS (1.000)	SS (1.000)
SS 17	SS (0.779)	SS (0.813)	SS (0.986)
SS 20	SS (0.907)	SS (1.000)	**NSS** (**0**.**266**)
SS 21	**NSS** (**0**.**132**)	**NSS** (**0**.**200**)	SS (1.000)
SS 22	SS (0.962)	SS (1.000)	SS (1.000)
SS 23	SS (0.970)	SS (1.000)	SS (1.000)
SS 24	SS (0.607)	SS (0.512)	**NSS** (**0**.**349**)
Accuracy	75%	86%	86%

## Discussion

Complex behaviors with common action patterns or modules, albeit in exaggerated or in compressed forms, can be precluded from identification as different. To address this issue we investigated two new applications of known techniques in computational geometry for curve alignment, which we call the Behavioral Distortion Distance or BDD, and for surface alignment that we call the Conformal Spatiotemporal Distance or CSD. These applications were motivated by the aim of devising generic methods that can be used to detect differences in a locomotory pattern without the need for experimenters to define the action sequence that constitutes a particular behavioral state. We applied them here to reexamine a report of an alarm response in medaka^36^. As the pattern of change in locomotion when a fish is alarmed is not universal and can vary in a species or context dependent manner^38,39^, it serves as a good test case for the two applications. We find that both BDD and CSD are suitable for quantitative comparison of such behaviors without the need to rely on domain specific expert knowledge of the underlying ethology. They confirm that indeed medaka behavior changes after exposure to *Schreckstoff* derived from conspecifics. The methods section describes the mathematical framework and definitions applied to develop these applications in detail.

The section analyzing pairwise differences shows that the fish in the two groups have similar locomotory behavior in the first 2-minutes, or in the pre-stimulus period. Therefore, there is no intrinsic behavioral difference between the groups at the onset. A significant change in BDD and CSD between the pre- and post-stimulus time intervals is noticeable only when the pairs had at least one fish exposed to *Schreckstoff*. This can be visualized more clearly in Figs 2 and 3. In such cases, the changes in BDD and CSD were complementary in the sense that the BDD was larger and the CSD was smaller. Notably, we did not observe significant differences in BDD between pairs of experimental fish and mixed pairs with one control fish and one experimental fish. This suggests that the behaviors of pairs of alarmed fish are as different from one another as they are from fish exhibiting normal behavior. In particular, BDD did not identify a stereotypical alarm response. While the CSD between mixed pairs was not significantly different from that of control pairs and experimental pairs, the mean CSD between experimental pairs was significantly lower than control (in fact, all) pairs. This suggests that alarmed fish exhibited a lower level of variability in their action pattern. One possible interpretation for this, following a visual inspection of the heatmaps of these individuals, is that this reduced variability could be due to periods of immobility represented as hot spots in their heatmaps.

Our analysis of the medaka alarm response using BDD and CSD brings together two seemingly contrasting ideas of stereotypic innate behavior and individual variability. Combining our observations, we conclude that the alarm response to *Schreckstoff* in medaka comprises increased variation in the local geometry and kinematics of a trajectory with a common spatiotemporal pattern. This is consistent with the ethogram of periods of erratic swimming interspersed with periods of immobility. Increased variation in the local geometry suggests swimming trajectories become less predictable over time in alarmed fish that likely serves as an advantageous anti-predator strategy. Yet, the ability of CSD to capture a commonality mirrors a description in Schleidt^5^ of “observer’s eye and mind perform a complex task of averaging and assigning weights to many different characteristics”, essentially integrating information over the experimental time period.

Following our analysis of pairwise BDD and CSD, we investigated the ability of Intra-Individual BDD (IIBDD, the average BDD from a subject to itself over many randomly sampled non-overlapping time intervals) to classify normal and alarmed behaviors. We found that IIBDD performed comparably in this task to the duration of immobility via logistic regressions.

**Figure 4:**
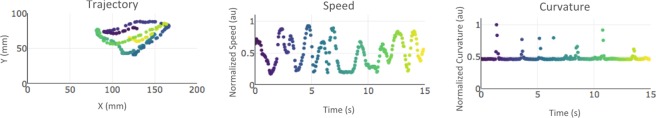
The trajectory, speed, and curvature plots of the first 15 seconds of the fish NSS 01. The same color scale is used in each plot so corresponding points on each curve are colored with the same color.

For our implementation of BDD, we took into account kinematic factors of medaka swimming such as the instantaneous speed (rate of displacement), as well as intrinsic geometric properties of the trajectory such as curvature (the rate of change of turning while traveling at unit speed; see Fig. 4 and Supplementary Video S1). Our choice of these factors was dependent on the information available in the original data-set, however, additional factors such as posture, if available, can be easily incorporated to calculate the distance between two generic behavioral curves. When comparing two behavioral curves, multiple optimal solutions are possible. In our context, such cases are considered equivalent. For instance, if we compare an episode in which an animal turned exactly once to an episode where a second animal turned exactly twice with the same curvature for each turn and speed, there will be two minima for BDD - aligning the single spike in curvature from the first episode to either of the two spikes in curvature from the second episode. Both these possible alternative alignments will give the same BDD score. We consider the two alternative alignments to be equivalent as they do not reveal any additional information about the behavior of the two animals other than the fact that the two episodes exhibited different behaviors.

A general caveat of using the BDD and CSD for analysis is that they can only be applied to a pair of curves (resp. heat maps) at a time. It therefore accompanies a degree to difficulty in visualizing a characteristic feature, if any, common to all individuals when comparing two conditions. As demonstrated here, it is possible to overcome this limitation to an extent by (1) statistical analysis of all pairs and (2) intra-individual comparisons. Another caveat of these measures is the assumption that the behavior being analyzed can be described by the intrinsic geometric and kinematic properties of locomotion. Indeed, behavioural posture is unaccounted in these analyses. However, here this limitation exists because of the recording technique employed. It can be overcome if postural data is identified in videos with higher resolution recordings that could then be incorporated to compute the BDD and CSD. In medaka, a higher BDD value and a lower CSD value represented an alarmed state. This may not be a characteristic feature of alarmed states in all species. However, if there is any substantial change in the pattern of locomotion compared to normal state in a species, it will likely be revealed in a change in BDD and CSD values in spite of individual differences in the execution of the motor action.

More generally, we have implemented the curve alignment process so that it can be used to compare any two curves that change over a common length of time. Our implementation can generate parametric curves from tracking data normally obtained for analysis of behavior (that is, *x*- and *y*-coordinates, time-stamp, frame rate, etc.) These new applications can therefore be used to compare locomotion dependent behavior of any animal tracked in a two dimensional arena. They will be particularly useful for characterizing the degree of similarity when comparing two or more conditions - such as when different treatments of a drug are applied to different groups, or in studying differences in exploratory pattern in different groups of animals. As demonstrated above, another useful aspect of these measures is that once a data-set is generated, the IIBDD from a smaller subset in a subsequent experiment is also informative provided the conditions of the original experiment are replicated when generating new data. These applications can also be applied to animals moving in 3-dimensional arena. We expect these methods will be more widely used after this demonstration of their utility. Accordingly, our software package is publicly available at the GitHub repository: https://github.com/mechunderlyingbehavior/locomotion.git.

## Methods

### Behavioral data

The data used for testing the applications comes from Mathuru^36^. Details of the experimental setup, including the method for preparation of the alarm substance (*Schreckstoff*), can be found in the methods section of the original publication. Briefly, fish behavior was recorded on an external Agent v5 HD web camera placed approximately 50 cm in front of the tank. Recorded videos were digitized at 20 frames/second. The frames were converted to 8-bit grayscale thresholded images by subtracting the background on ImageJ (from https://imagej.nih.gov/ij/). The spatial resolution of the videos was 2.4 pixels/mm. Fish position was tracked automatically on MetaMorph 6.3 (from https://www.moleculardevices.com) using the “track objects” algorithm. The original tracking data used in our model is uploaded to public data repository and is provided with this paper. Experiments in the original dataset were performed in accordance to the guidelines recommended by the Institutional Animal Care and Use Committee (IACUC) of the Biological Resource Center at A*STAR. Approved experimental protocols (IACUC 161110) were followed.

### Curve alignment and behavioral distortion distance (BDD)

Our first method compares the behavior of two animals by comparing the intrinsic geometric properties and dynamics of their trajectories. It is an application of the *edit distance* between curves introduced by Sebastian *et al*.^16^, which can be calculated via a technique known as *dynamic time warping* introduced by Sakoe and Chiba^20^.

#### Theoretical framework and definitions

We assume that the trajectory of each fish is a continuous, smooth parametric curve given by a vector-valued function ${\bf{r}}:[{t}_{0},{t}_{f}]\to {{\mathbb{R}}}^{3}$ where *t*_0_ and *t*_*f*_ denote the start and end times of a specified time interval, respectively. To identify the geometric and dynamic features of the trajectories that are most relevant to behavior, we first calculate the rate at which an animal’s motion is changing relative to its current frame of reference. We model an animal’s frame of reference at a point along its trajectory with its *Frenet-Serret frame* at that point: For a non-degenerate, continuously differentiable, parametric curve *C* with the vector-valued function ${\bf{r}}:[{t}_{0},{t}_{f}]\to {{\mathbb{R}}}^{3}$ it is well known in differential geometry^27^ that the unit tangent, normal, binormal vectors, defined by

$$T(t)=\frac{{\bf{r}}^{\prime} (t)}{||{\bf{r}}^{\prime} (t)||},\,N(t)=\frac{T^{\prime} (t)}{||T^{\prime} (t)||},\,{\rm{and}}\,B(t)=T(t)\times N(t),$$

respectively, at each point of the trajectory form an orthonormal basis of ${{\mathbb{R}}}^{3}$. (Here, *g*′(*t*) denotes the derivative of a function *g* with respect to *t*, ||**v**|| denotes the magnitude of a vector ${\bf{v}}\in {{\mathbb{R}}}^{3}$, and × denotes the cross product.) In other words, we can recoordinatize ${{\mathbb{R}}}^{3}$ according to a fish’s position and orientation at each moment in time. Measuring the rate of change of an animal’s frame of reference at a given point is equivalent to calculating the rate of change of its Frenet-Serret frame, given by the famous Frenet-Serret formula^51,52^, which states that if ${\bf{r}}:[{t}_{0},{t}_{f}]\to {{\mathbb{R}}}^{3}$ is a non-degenerate, continuously differentiable, parametric curve and *T*(*t*), *N*(*t*), and *B*(*t*) are its unit tangent, normal, and binormal vectors at time *t*, then

$$\frac{d}{dt}\,[\begin{array}{c}T(t)\\ N(t)\\ B(t)\end{array}]=s(t)\,[\begin{array}{ccc}0 & \kappa (t) & 0\\ -\kappa (t) & 0 & \tau (t)\\ 0 & -\tau (t) & 0\end{array}]\,[\begin{array}{c}T(t)\\ N(t)\\ B(t)\end{array}]$$

where

$$s(t)=\Vert {{\bf{r}}}^{{\rm{^{\prime} }}}(t)\Vert ,\,\kappa (t)=\frac{||{{\bf{r}}}^{{\rm{^{\prime} }}}(t)\times {{\bf{r}}}^{{\rm{^{\prime} }}{\rm{^{\prime} }}}(t)||}{||{{\bf{r}}}^{{\rm{^{\prime} }}}(t{)||}^{3}},\,{\rm{a}}{\rm{n}}{\rm{d}}\,\tau (t)=\frac{det({{\bf{r}}}^{{\rm{^{\prime} }}}(t),{{\bf{r}}}^{{\rm{^{\prime} }}{\rm{^{\prime} }}}(t),{{\bf{r}}}^{{\rm{^{\prime} }}{\rm{^{\prime} }}{\rm{^{\prime} }}}(t))}{||{{\bf{r}}}^{{\rm{^{\prime} }}}(t)\times {{\bf{r}}}^{{\rm{^{\prime} }}{\rm{^{\prime} }}}(t{)||}^{2}}.$$

The upshot of the Frenet-Serret formula in our context is that the rate of change of the Frenet-Serret frame is completely determined by *s*(*t*), *κ*(*t*), and *τ*(*t*), which are the *speed*, *curvature*, and *torsion*, respectively, of **r** at time *t*. While speed will be familiar to many readers as the rate of displacement, the curvature indicates the rate at which the curve is turning while traveling at unit speed, and the torsion indicates the rate which the curve is twisting while traveling at unit speed. Portions of the trajectory, speed, and curvature of the Medaka NSS 01 fish (Supplementary Video 1) over the first fifteen seconds of its experiment are shown in Fig. 4 and in Supplementary Video 2.

Depending on the application, there may be additional factors that are relevant to behavior, such as position, posture, or distance to an object of interest. To accomodate such factors, we introduce the following notion:

##### **Definition 1.**

 Let ${\bf{r}}\,:[{t}_{0},{t}_{f}]\to {{\mathbb{R}}}^{3}$ be the trajectory of an animal and let $\Theta =\{{\theta }_{1},\ldots ,{\theta }_{m}\}$ be a set of *m* behavioral factors, such as *s*, *κ*, and *τ*. The behavior curve of the trajectory indexed by Θ is the parametric curve ${C}_{\Theta }\,:[{t}_{0},{t}_{f}]\to {{\mathbb{R}}}^{m}$ given by ${C}_{\Theta }(t)=({\theta }_{1}(t),\ldots ,{\theta }_{m}(t))$.

Our objective is to find an acceptable notion of distance between the behavior curves of two episodes. Because it is virtually impossible to deliver an external chemical stimulus simultaneously to two freely swimming animals (as the stimulus takes time to disperse through the tank water), it is not meaningful to compare two behavior curves at common moments in time. For instance, a slight time lag due to different reaction times could yield arbitrarily large quantitative differences between two animals that exhibit identical sequences of behaviors with that approach. Instead, we must identify an appropriate correspondence between pairs of points from each behavior curve in order to make a meaningful comparison. To solve this curve alignment problem, we apply the *edit distance* developed by Sebastian *et al*.^16^, which measures the least amount of energy required to bend, stretch, and/or compress one parametric curve onto another by finding an alignment that minimizes a specified energy. In our context, an *alignment* between two parametric curves ${C}^{1}\,:[{t}_{0},{t}_{f}]\to {{\mathbb{R}}}^{{d}_{1}}$ and ${C}^{2}\,:[{t}_{0}^{\ast },{t}_{f}^{\ast }]\to {{\mathbb{R}}}^{{d}_{2}}$ is a function

$$\phi \,:[{u}_{0},{u}_{f}]\to [{t}_{0},{t}_{f}]\times [{t}_{0}^{\ast },{t}_{f}^{\ast }]\,{\rm{with}}\,\phi (u)=({\phi }_{1}(u),{\phi }_{2}(u))$$

satisfying the conditions that $\phi ({u}_{0})=({t}_{0},{t}_{0}^{\ast })$, $\phi ({u}_{f})=({t}_{f},{t}_{f}^{\ast })$, and *φ*_*i*_ is monotone increasing and differentiable for each *i* ∈ {1, 2}, and the *energy* we seek to minimize is given by

$$E(\phi )=\frac{1}{{u}_{f}-{u}_{0}}{\int }_{{u}_{0}}^{{u}_{f}}\sqrt{\mathop{\sum }\limits_{i=1}^{d}\,{({C}_{i}^{1}({\phi }_{1}(u))-{C}_{i}^{2}({\phi }_{2}(u)))}^{2}}\,du.$$

We can now define the behavioral distortion distance:

##### **Definition 2.**

 Let ${{\bf{r}}}_{1}\,:[{t}_{0},{t}_{f}]\to {{\mathbb{R}}}^{d}$ and ${{\bf{r}}}_{2}\,:[{t}_{0}^{\ast },{t}_{f}^{\ast }]\to {{\mathbb{R}}}^{d}$ be the trajectories of two animals and let $\Theta =\{{\theta }_{1},\ldots ,{\theta }_{m}\}$ be a set of behavioral factors. The behavioral distortion distance between **r**_1_ and **r**_2_ with respect to Θ is

$${{\rm{BDD}}}_{\Theta }({{\bf{r}}}_{1},{{\bf{r}}}_{2})=E({\phi }_{{\rm{\min }}})$$

where *φ*_min_ is an alignment between the behavior curves ${C}_{\Theta }^{1}$ and ${C}_{\Theta }^{2}$ corresponding to **r**_1_ and **r**_2_, respectively, that minimizes *E*.

**Figure 5:**
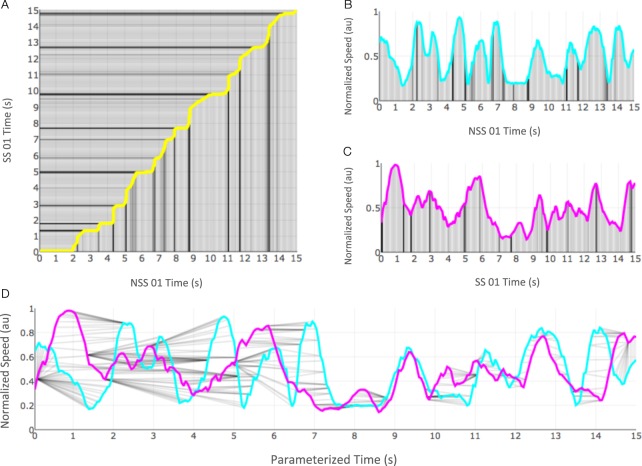
An alignment between the speed curves of the NSS 01 and SS 01 medaka over the first 15 seconds of the experiment. (**A**) The optimal alignment *φ*_min_; (**B**) the speed curve for NSS 01; (**C**) the speed curve for SS 01; (**D**) the two curves plotted simultaneously with pairs of matched points indicated by gray line segments; (**E**) the two curves plotted with parameterized or “warped” time.

An example optimal alignment between the behavior curves with Θ = {*s*} for the first 15 seconds of observation for the Medaka NSS 01 fish and Medaka SS 01 fish is illustrated in Fig. 5. Supplementary Video 3 is an animation example that illustrates this alignment over a 30 second observation interval of the same pair of animals.

#### Implementation

We obtain the trajectories from the “track objects” method in the MetaMorph software as sequences of *x*- and *y*-coordinates in the real plane. While the observational tank is 3-dimensional, the camera only captures the projection of a 2-dimensional, 20 cm × 12 cm, vertical cross-section of an animals movement. As described in Mathuru^36^, since videos were recorded from the front of the tank, displacement in the orthogonal width could not be recorded, effectively reducing a three dimensional space to a two dimensional observation. Ideally, we would be able to capture 3-dimensional trajectories; however, the BDD has the same derivation for 2-dimensional trajectories. The Frenet-Serret frames of a plane curve are given by the tangent and normal vectors and the Frenet-Serret formula is identical with torsion constantly equal to 0.

**Figure 6:**
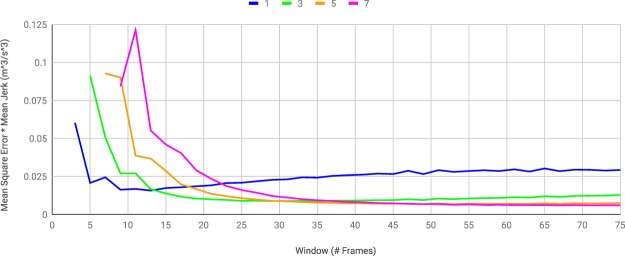
Products of mean square error and mean jerk of Savitzky-Golay smoothing with respect to window length for each odd order between 1 and 7. The plots for the pairs of orders 2 and 3, 4 and 5, and 6 and 7 were indistinguishable in the graph, so the even orders were omitted.

We use *dynamic time warping* (DTW) to calculate BDD, the same approach Sebastian *et al*.^16^ use to calculate edit distance. Our implementation of the DTW algorithm is an extension of the standard DTW method in the Machine Learning module, mlpy^53^, in Python (see Muller^54^ for a description of how DTW works). Specifically, we modified the existing DTW method in mlpy to compare multivariate time series (instead of only univariate time series) by changing the distance function from 1-dimensional Euclidean distance to *m*-dimensional Euclidean distance for a specified *m*. While our method is able to handle input data of an arbitrarily high dimension (any number of behavioral factors), the analyses in this study have only used speed and curvature. The speed and curvature values are generated from the *x*- and *y*-coordinates using the formulas

$$s(t)=\sqrt{x^{\prime} {(t)}^{2}+y^{\prime} {(t)}^{2}}\,{\rm{and}}\,\kappa (t)=\frac{|x^{\prime} (t)y^{\prime\prime} (t)-y^{\prime} (t)x^{\prime\prime} (t)|}{v{(t)}^{3}},$$

where the derivatives are computed by applying the gradient function in the NumPy^55^ Python package to the *x* and *y* time series. We smoothed the trajectories using the Savitzky-Golay function in the SciPy^56^ Python package (see Savitzky and Golay^57^ for a description of the Savitzky-Golay smoothing filter). That function takes two integer parameters, namely the order of the polynomials used for smoothing and length (number of frames) of the smoothing window centered at each frame. Since the choice of parameters is application dependent, we searched for the parameters that simultaneously minimized mean square error (deviation from raw trajectory) and jerk (rate of change of acceleration) for every order between 1 and 9 and every window length up to 600 frames (30 seconds). We encountered numerical instability for orders above 7. The products of mean square error and mean jerk with respect to window length are plotted for each order between 1 and 7 in Fig. 6. We used the order 5 Savitzy-Golay smoothing with a window length of 53 frames.

Furthermore, to account for variations in mean values of behavioral factors between animals and differences in magnitudes between behavioral factors themselves, we normalize all of the data using the sigmoid function

$${\rm{sig}}\,(x)=\frac{1}{1+{e}^{-(x-\mu )/\sigma }},$$

where *μ* and *σ* are the mean and the standard deviation of the respective input sequence. We chose the sigmoid function for our particular application because the distribution of the curvatures of the trajectories is positively skewed, as can be seen from Fig. 4. Since the range of the sigmoid function is [0, 1], the value of BDD_Θ_ is bounded above by the square root of the number of factors in Θ.

### Surface alignment and conformal spatiotemporal distance (CSD)

The second application we considered compares the spatiotemporal distributions between two animals. When comparing heat maps of time spent at each location in an arena between animals, it is not useful to look at a straightforward average since hot spots from different animals will average out and disappear. Instead, one must first align the heat maps so corresponding hot spots coincide in order to reveal if there are common patterns and similarities. To do this, we apply the *symmetric distortion energy* between surfaces introduced by Koehl and Hass^30,58^.

#### Theoretical framework and definitions

**Figure 7:**
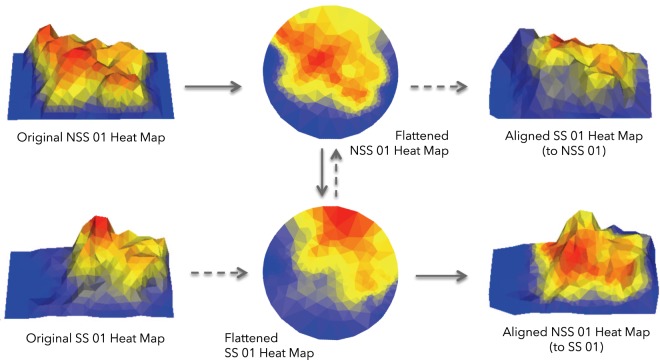
The approximately uniform Delaunay triangulations corresponding to the heat maps of the NSS 01 and SS 01 medaka, their conformal flattenings to the unit disk, the symmetric distortion minimizing Möbius transformation from the unit disk to itself, and corresponding alignments.

To examine the overall spatiotemporal patterns of an animal within an enclosure, we study its spatiotemporal distribution function, that is, the continuous function $\rho :\Omega \to {\mathbb{R}}$ where Ω is the enclosure of the animal and *ρ*(*z*) is the number of seconds the animal spends at location *z* within Ω over a specified time interval *I*. When Ω is a region in the plane, this function can be visualized/interpreted as a heatmap where small values of *ρ* correspond to “cold” points and large values of *ρ* correspond to “hot” points. Alternatively, the spatiotemporal distribution function can be visualized/interpreted as the surface in ${{\mathbb{R}}}^{3}$ with points {(*x*, *y*, *ρ*(*x*, *y*)|(*x*, *y*) ∈ Ω)}, as illustrated in Fig. 7. We assume that this surface is smooth and Riemannian, that is, it is equipped with a well-defined notion of distance and angles.

We can compare the spatiotemporal distribution functions between two animals by taking an appropriate norm between them, but that will be sensitive to a variety of factors that cannot be controlled, such as the position of an animal when the substance is administered. Mirroring our formulation of BDD, we must build some flexibility into our model by first aligning the corresponding surfaces before calculating a norm between them.

To align a pair of Riemannian surfaces *S*_1_ and *S*_2_, we look at the space of diffeomorphisms between them. A differentiable map *f*: *S*_1_ → *S*_2_ is a *diffeomorphism* if it is a bijection and its inverse *f  *^−1^ is also differentiable. A diffeomorphism gives a point-to-point correspondence between surfaces in a way that does not introduce any tears or folds, but it can distort the shape of a surface in unusual ways, for instance by stretching it. Ideally, we would like to find a diffeomorphism that does not distort *S*_1_ while mapping it onto *S*_2_; such a mapping is called an *isometry*. Unfortunately, an isometry between *S*_1_ and *S*_2_ may not exist. Any diffeomorphism between surfaces with different areas, for instance, will have to stretch one of the surfaces somewhere. While we cannot hope to find an isometry between *S*_1_ and *S*_2_, we can always find a map that preserves angles, such a map is called *conformal*. This is the content of a deep result in geometry called the *Uniformization Theorem*^59^, which states that every simply connected Riemann surface is conformally equivalent to one of three Riemann surfaces: the open unit disk, the complex plane, or the Riemann sphere. While a conformal mapping *f* : *S*_1_ → *S*_2_ may distort distances, it will stretch *S*_1_ uniformly in all directions at each point. This defines a function ${\lambda }_{f}:{S}_{1}\to {\mathbb{R}}$ that maps each point *z* in *S*_1_ to the factor by which vectors at *p* are stretched by *f*. The map *f* is an isometry if and only if *λ*_*f*_(*z*) = 1 for all *z* ∈ *S*_1_. Accordingly, one can measure how far a conformal diffeomorphism is from being an isometry be calculating how far the stretching factor is from 1 at each point. Koehl and Hass^58^ introduced the *symmetric distortion energy* of a conformal diffeomorphism *f* : *S*_1_ → *S*_2_ with dilation functions ${\lambda }_{f}\,:{S}_{1}\to {\mathbb{R}}$ and ${\lambda }_{{f}^{-1}}\,:{S}_{2}\to {\mathbb{R}}$ as

$${E}_{sd}(f)=\sqrt{{\int }_{{S}_{1}}\,{({\lambda }_{f}(z)-1)}^{2}\,dA}+\sqrt{{\int }_{{S}_{2}}\,{({\lambda }_{{f}^{-1}}(z)-1)}^{2}\,dA}.$$

In our context, an optimal alignment between *S*_1_ and *S*_2_ will be a conformal diffeomorphism that minimizes the symmetric distortion energy. We can then measure the difference between two aligned surfaces by calculating the *L*_2_-norm between their corresponding functions.

##### **Definition 3.**

Let *S*_1_ and *S*_2_ be the surfaces of spatiotemporal distribution functions ${\rho }_{1}\,:\Omega \to {\mathbb{R}}$ and ${\rho }_{2}\,:\Omega \to {\mathbb{R}}$, respectively, and for every diffeomorphism *f* : *S*_1_ → *S*_2_ and $z\in \Omega$ let *z*_*f*_ denote the unique point such that $f((z,{\rho }_{1}(z)))=({z}_{f},{\rho }_{2}({z}_{f}))$. The *conformal spatiotemporal distance* between *S*_1_ and *S*_2_ is given by

$$\text{CSD}({S}_{1},{S}_{2})=\frac{1}{2||\Omega ||}(\sqrt{{\int }_{\Omega }{({\rho }_{1}(z)-{\rho }_{2}({z}_{{f}_{\ast }}))}^{2}\,dA}+\sqrt{{\int }_{\Omega }{({\rho }_{2}(z)-{\rho }_{1}({z}_{{f}_{\ast }^{-1}}))}^{2}\,dA}\,)$$

where $||\Omega ||$ denotes the area of Ω and ${f}_{\ast }\,=\,{{\rm{argmin}}}_{{\rm{conformal}}f}{E}_{sd}(f)$.

#### Implementation

To approximate the spatiotemporal distribution function for each fish, we subdivide the rectangular cross-section of the experimental enclosure into an *m* × *n* rectangular grid and assign to each subrectangle the percentage of time spent in that region throughout a specified duration of time. We model the surface corresponding to this function by triangulating each of the rectangular regions and taking the induced triangulation on the corresponding set of points in ${{\mathbb{R}}}^{3}$. To approximate a conformal mapping from this surface to the unit disk, it will be helpful to have a regular or uniform (all of the edge lengths are approximately equal) triangulation of the surface. Accordingly, we subdivide the edges of our preliminary triangulated surface so that all of the edge lengths are within a specified tolerance of equality and apply the Bowyer-Watson algorithm^60,61^ to obtain a Delaunay triangulation of the resulting vertex set.

We apporixmate the set of conformal mappings between two such triangulations with the Discrete Uniformization theorem^62^. In particular, we use an algorithm developed by Collins and Stephenson^63^ to find a discrete conformal mapping ${f}_{i}:{S}_{i}\to {\mathbb{D}}$ from each triangulated surface / heat map to a triangulation of the unit disk. Once we have these conformal flattening maps, *f*_1_ and *f*_2_, we can express every conformal mapping *f* : *S*_1_ → *S*_2_ as $f={f}_{2}^{-1}\circ \,m\,\circ \,{f}_{1}$ where $m\,:{\mathbb{D}}\to {\mathbb{D}}$ is a *Möbius transformation* of the unit disk. Finding an optimal alignment between *S*_1_ and *S*_2_ is then reduced to finding a Möbius transformation $m\,:{\mathbb{D}}\to {\mathbb{D}}$ whose composition ${f}_{2}^{-1}\circ m\circ {f}_{1}$ minimizes the symmetric distortion energy. This is illustrated in Fig. 7.

A well known application of Schwarz’s Lemma^64^ in complex analysis is that every Möbius transformation from ${\mathbb{D}}$ to itself is determined by which point is sent to the origin and a rotation. For stability reasons, we map the centroid of each surface to the origin^58^. This also reduces the minization problem to a single rotation, which we approximate by brute force, although there are algorithms in the literature for this^29^. We use the following discrete analogue of the symmetric distortion energy presented in Koehl and Hass^58^: If *T*_1_ and *T*_2_ are triangulations of smooth Riemannian surfaces *S*_1_ and *S*_2_, respectively, and *f* : *S*_1_ → *S*_2_ is a conformal mapping, then

$${E}_{sd}(f)\approx \sqrt{\sum _{{e}_{ij}\in {T}_{1}}\,{(\frac{\ell (f({e}_{ij}))}{\ell ({e}_{ij})}-1)}^{2}\frac{{A}_{ij}}{3}}+\sqrt{\sum _{{e}_{ij}\in {T}_{2}}\,{(\frac{\ell ({f}^{-1}({e}_{ij}))}{\ell ({e}_{ij})}-1)}^{2}\frac{{A}_{ij}}{3}}$$

where *e*_*ij*_ denotes an edge in *T*_1_ or *T*_2_, $\ell$ denotes the length of an edge (or its image), and *A*_*ij*_ is the sum of the areas of the two triangles that contain the edge *e*_*ij*_. Here the sums run over all of the interior edges in *T*_1_ and *T*_2_ respectively.

### Statistical analyses

All of the Kruskal-Wallis H, Mann-Whitney U, and Wilcoxon T tests presented in the Results section were executed in SciPy^56^. A p value of 0.01 was considered significant. When multiple comparisons were made Bonferroni correction for multiple comparisons was applied to determine statistial significance. When reporting, the P values up to the third decimal place are presented instead of an asterisk sign.

## Supplementary information

Supplementary Table S1

Supplementary Video S1

Supplementary Video S2

Supplementary Video S3
