# Supplementary material for: Computational geometric tools for quantitative comparison of locomotory behavior
Source: Sci Rep. 2019 Nov 12;9:16585. doi: 10.1038/s41598-019-52300-8 (PMC6851375; doi:10.1038/s41598-019-52300-8)
Supplement: Supplementary file 1 — Supplementary Table S1 [file 41598_2019_52300_MOESM1_ESM.pdf]

Computational geometric tools for quantitative comparison of locomotory behavior

Matthew T. Stamps[1], **Soo Go**[1,†], **and Ajay S. Mathuru**[1,2,3,*]

[1]Yale-NUS College, Singapore
[2]Institute of Molecular and Cell Biology (IMCB), Singapore
[3]Department of Physiology, Yong Loo Lin School of Medicine (YLL), National University of Singapore, Singapore
[†]At the time the work was performed.
[*]ajay.mathuru@yale-nus.edu.sg

ABSTRACT

A fundamental challenge for behavioral neuroscientists is to accurately quantify (dis)similarities in animal behavior without excluding inherent variability present between individuals. We explored two new applications of curve and shape alignment techniques to address this issue. As a proof-of-concept we applied these methods to compare normal or alarmed behavior in pairs of medaka (*Oryzias latipes*). The curve alignment method we call Behavioral Distortion Distance (BDD) revealed that alarmed fish display less predictable swimming over time, even if individuals incorporate the same action patterns like immobility, sudden changes in swimming trajectory, or changing their position in the water column. The Conformal Spatiotemporal Distance (CSD) technique on the other hand revealed that, in spite of the unpredictability, alarmed individuals share an overall swim pattern, possibly accounting for the widely held notion of "stereotypy" in alarm responses. More generally, we propose that these new applications of established computational geometric techniques are useful in combination to represent, compare, and quantify complex behaviors consisting of common action patterns that differ in duration, sequence, or frequency.

Supplementary Table

The following Supplementary Table 1 is referred to in the results section.

Subject	Average Speed (m/s)	Immobility (s)	IIBDD (au)	NSSBDD (au)	SSBDD (au)
NSS 01	35.221	1.50	0.074	0.075	0.121
NSS 02	34.024	1.75	0.089	0.081	0.125
NSS 03	19.534	12.25	0.084	0.086	0.124
NSS 04	34.133	4.00	0.078	0.076	0.126
NSS 05	19.664	14.75	0.083	0.086	0.123
NSS 06	34.480	0.00	0.074	0.075	0.123
NSS 07	31.317	2.00	0.071	0.074	0.124
NSS 08	47.490	0.75	0.065	0.075	0.128
NSS 09	37.287	0.50	0.071	0.074	0.126
NSS 10	33.041	1.75	0.065	0.073	0.124
NSS 11	37.016	0.00	0.064	0.073	0.125
NSS 12	24.291	3.50	0.084	0.081	0.123
NSS 13	24.337	8.00	0.080	0.080	0.123
NSS 14	25.985	6.75	0.091	0.084	0.125
NSS 15	38.479	0.25	0.071	0.082	0.131
NSS 16	39.102	1.75	0.079	0.076	0.125
NSS 17	30.703	0.75	0.075	0.075	0.122
NSS 18	38.507	0.75	0.081	0.076	0.123
SS 01	26.352	30.25	0.103	0.134	0.096
SS 03	15.401	76.00	0.093	0.129	0.083
SS 04	29.454	14.50	0.098	0.132	0.093
SS 05	15.527	34.00	0.123	0.154	0.127
SS 06	7.534	97.25	0.087	0.129	0.086
SS 07	23.213	16.50	0.092	0.129	0.084
SS 08	28.823	20.00	0.082	0.127	0.082
SS 09	17.648	18.50	0.085	0.130	0.080
SS 10	37.368	5.25	0.085	0.129	0.086
SS 14	19.761	10.00	0.087	0.130	0.087
SS 15	18.899	30.50	0.105	0.136	0.098
SS 16	15.099	45.50	0.124	0.148	0.113
SS 17	17.752	84.00	0.102	0.138	0.106
SS 20	12.919	49.50	0.081	0.129	0.078
SS 21	37.745	5.50	0.148	0.160	0.132
SS 22	7.203	85.75	0.286	0.314	0.302
SS 23	5.764	102.75	0.276	0.438	0.431
SS 24	23.927	10.25	0.082	0.128	0.079

Table S 1. The average speed, duration of immobility, intra-individual BDD (IIBDD), mean BDD of the subject to all control subjects (NSSBDD), and to all experimental subjects (SSBDD)
